# Supplementary material for: MetaQTL: a package of new computational methods for the meta-analysis of QTL mapping experiments
Source: BMC Bioinformatics. 2007 Feb 8;8:49. doi: 10.1186/1471-2105-8-49 (PMC1808479; doi:10.1186/1471-2105-8-49)
Supplement: Additional File 7 — MetaQTL Package : jar file and tutorial. This Zip archive contains both the MetaQTL JAR file and the files of the tutorial. [file 1471-2105-8-49-S7.zip › org.inra.metaqtl/doc/org/metaqtl/MetaChrom.html]

MetaChrom


|  |  |  |
| --- | --- | --- |
| **Overview** \| **Package** \| **Class** \| **Use** \| **Tree** \| **Deprecated** \| **Index** \| **Help** | |  |
| **PREV CLASS**   **NEXT CLASS** | **FRAMES**    **NO FRAMES**     **All Classes** |  |
| SUMMARY: NESTED \| FIELD \| CONSTR \| METHOD | DETAIL: FIELD \| CONSTR \| METHOD |  |


---


## org.metaqtl Class MetaChrom

```
java.lang.Object
  org.metaqtl.MetaChrom
```

---

``` public class MetaChrom extends java.lang.Object ```

A MetaChrom is the result of the merge of the members of a
`ChromCluster`.

---

| **Field Summary** | |
| --- | --- |
| `double` | `chi2`             The chi-square value of this chromosome. |
| `double[]` | `d`             The absolute positions of markers in Morgan. |
| `int` | `dof`             The number of degrees of freedom. |
| `java.lang.String[]` | `mapNames`             The names of the genetic maps. |
| `java.lang.String[]` | `mrkNames`             The ordered marker names. |
| `int[]` | `mrkOc`             The number of occurence per marker oved chromosomes. |
| `java.lang.String` | `name`             The name of the meta chrom. |
| `int` | `nc`             The number of chromosomes implied. |
| `int` | `ni`             The number of marker intervals implied in this consensus. |
| `int` | `nm`             The number of markers. |
| `int[]` | `nmc`             The number of marker per implied chromosome |
| `double` | `pvalue`             The p-value of this chromosome. |
| `double[]` | `rsd`             The standardized residual between original marker intervals and those of the meta chromosome. |
| `double[]` | `sdd`             The standard deviation of the position in Morgan. |


**Constructor Summary**

`MetaChrom(MultiFitResult result,
ChromCluster chromCluster)`
  
          Get a new instance of `MetaChrom` which is
initialized by using the result
of the merge of the members of a `ChromCluster`.


**Method Summary**

**Methods inherited from class java.lang.Object**

`clone, equals, finalize, getClass, hashCode, notify, notifyAll, toString, wait, wait, wait`

**Field Detail**

### nc

```
public int nc
```

:   The number of chromosomes implied.

---


### nm

```
public int nm
```

:   The number of markers.

---


### ni

```
public int ni
```

:   The number of marker intervals implied in this consensus.

---


### dof

```
public int dof
```

:   The number of degrees of freedom.

---


### nmc

```
public int[] nmc
```

:   The number of marker per implied chromosome

---


### mrkOc

```
public int[] mrkOc
```

:   The number of occurence per marker oved chromosomes.

---


### chi2

```
public double chi2
```

:   The chi-square value of this chromosome.

---


### pvalue

```
public double pvalue
```

:   The p-value of this chromosome.

---


### rsd

```
public double[] rsd
```

:   The standardized residual between original marker intervals
    and those of the meta chromosome.

---


### d

```
public double[] d
```

:   The absolute positions of markers in Morgan.

---


### sdd

```
public double[] sdd
```

:   The standard deviation of the position in Morgan.

---


### name

```
public java.lang.String name
```

:   The name of the meta chrom.

---


### mapNames

```
public java.lang.String[] mapNames
```

:   The names of the genetic maps.

---


### mrkNames

```
public java.lang.String[] mrkNames
```

:   The ordered marker names.


**Constructor Detail**

### MetaChrom

```
public MetaChrom(MultiFitResult result,
                 ChromCluster chromCluster)
```

:   Get a new instance of `MetaChrom` which is
    initialized by using the result
    of the merge of the members of a `ChromCluster`.

    **Parameters:**: `result` - the result of the merge of the members of the cluster.: `cluster` - the chromosome cluster.


---


|  |  |  |
| --- | --- | --- |
| **Overview** \| **Package** \| **Class** \| **Use** \| **Tree** \| **Deprecated** \| **Index** \| **Help** | |  |
| **PREV CLASS**   **NEXT CLASS** | **FRAMES**    **NO FRAMES**     **All Classes** |  |
| SUMMARY: NESTED \| FIELD \| CONSTR \| METHOD | DETAIL: FIELD \| CONSTR \| METHOD |  |


---
